# Downregulation of *GPR155* as a prognostic factor after curative resection of hepatocellular carcinoma

**DOI:** 10.1186/s12885-017-3629-2

**Published:** 2017-09-01

**Authors:** Shinichi Umeda, Mitsuro Kanda, Hiroyuki Sugimoto, Haruyoshi Tanaka, Masamichi Hayashi, Suguru Yamada, Tsutomu Fujii, Hideki Takami, Yukiko Niwa, Naoki Iwata, Chie Tanaka, Daisuke Kobayashi, Michitaka Fujiwara, Yasuhiro Kodera

**Affiliations:** Department of Gastroenterological Surgery (Surgery II), Nagoya University Graduate School of Medicine, 65 Tsurumai-cho, Showa-ku, Nagoya, 466-8550 Japan

**Keywords:** Hepatocellular carcinoma, *GPR155*, Expression, Recurrence, Biomarker

## Abstract

**Background:**

Molecular biomarkers capable of predicting recurrence patterns and prognosis are helpful for risk stratification and providing appropriate treatment to patients with hepatocellular carcinoma (HCC). In this study, we focused on G protein-coupled receptor 155 (*GPR155*), a cell surface signaling protein, as a candidate biomarker.

**Methods:**

We analyzed *GPR155* expression, DNA methylation, and copy number in HCC cell lines. The clinical significance of *GPR155* expression was evaluated using 144 pairs of surgically resected liver and normal tissues with subgroup analysis based on hepatitis virus infection.

**Results:**

*GPR155* mRNA expression levels were differential and were decreased in 89% of HCC cell lines. No DNA methylation was detected, whereas copy number alterations were present in five (56%) HCC cell lines. *GPR155* mRNA expression level was independent of background liver status and significantly lower in HCC tissues than corresponding normal liver tissues. The expression patterns of GPR155 protein by immunohistochemical staining were significantly associated with those of *GPR155* mRNA. Downregulation of *GPR155* was significantly associated with more aggressive HCC phenotypes including high preoperative α-fetoprotein, poor differentiation, serosal infiltration, vascular invasion, and advanced disease stage. Patients with downregulation of *GPR155* were more likely to have worse prognosis after curative resection irrespective of hepatitis virus infection. Patients who experienced extrahepatic (distant) recurrences had significantly lower *GPR155* expression than those with intrahepatic (liver confined) recurrences.

**Conclusions:**

Downregulation of *GPR155* may serve as a prognosticator that also predicts initial recurrence sites independent of hepatitis virus infection.

**Electronic supplementary material:**

The online version of this article (10.1186/s12885-017-3629-2) contains supplementary material, which is available to authorized users.

## Background

Hepatocellular carcinoma (HCC) ranks at the third most common cause of cancer-related death in the world [1, 2]. Although liver resection has been the mainstay of treatment for HCC, the recurrence rate after curative resection remains high at approximately 70% [2–4]. Complete cure of this disease is quite challenging even though various therapeutic modalities have been developed. A realistic initial goal is the establishment of methods for accurate risk stratification and prediction of recurrence sites after liver resection to provide appropriate perioperative management according to each individual patient’s circumstances [5]. The TNM classification system has been broadly employed as a tumor staging method to predict postoperative outcomes concisely but can be inaccurate [6, 7]. For example, patients with an earlier tumor stage sometimes have unfavorable prognosis. Extrahepatic recurrences, such as lung, bone, and brain metastases, can be a cause of an unexpected and rapidly deteriorating patient course; however, no methods for predicting the likelihood of extrahepatic recurrences of HCC are currently available [8, 9]. Conversely, some patients are long-term survivors after resection of advanced HCC without adjuvant therapy. To address these clinical issues, development of a novel molecular marker able to reflect potential characteristics of the tumor is required [10].

G protein-coupled receptors (GPCRs) are reportedly cell surface signaling proteins that have important roles in various physiological functions, and in initiation and progression of cancer [11]. The G protein-coupled receptor 155 gene (*GPR155*), present on 2q31.1, encodes a 97 kDa transmembrane receptor protein that is a member of the GPCR family [12]. Although there has been a report that *GPR155* expression is suppressed in neoplasms of the thyroid, the oncologic roles of *GPR155* in HCC remain unclear [13, 14]. We focus on *GPR155* because it is recognized as a transmembrane marker possibly associated with the transport of growth factors and anticancer drugs, and no published data of *GPR155* expression in HCC.

The aims of this study were to evaluate the clinical significance of *GPR155* expression, explore the factors that regulate *GPR155* transcription, and assess the performance of *GPR155* as a potential prognosticator of HCC.

## Methods

### Sample collection

Human HCC cell lines Hep3B, HepG2, PLC/PRF/5, and SK-Hep1, and the control nontumorigenic epithelial cell line FHs74 were obtained from the American Type Culture Collection (Manassas, VA). HLE, HLF, HuH1, and HuH7 cells were obtained from the Japanese Collection of Research Bioresources Cell Bank (Osaka, Japan). HuH2 was from Aichi Cancer Center (Nagoya, Japan). Primary HCC tissues and corresponding non-cancerous tissues were collected from 144 patients who underwent liver resection at Nagoya University Hospital between January 1998 and January 2012. All tissue samples were frozen immediately after resection and diagnosed histologically as HCC. Postoperative follow-up included physical examinations, measurement of serum tumor markers every 3 months, and enhanced computed tomography every 6 months [15]. Treatment after recurrence included surgery, radiofrequency ablation, transcatheter arterial chemoembolization, and chemotherapy, according to tumor status and liver function.

### Quantitative real-time RT-PCR (qRT-PCR)

Quantitative real-time reverse-transcription polymerase chain reaction (qRT-PCR) was used to determine the expression level of *GPR155* mRNA. Primer sequences are shown in Supplementary Table 1. Total RNA (10 μg per sample) was isolated from nine HCC cell lines, FHs74 cells, and 144 pairs of clinical samples and a quality check for all RNA samples was conducted before generating complementary DNAs (cDNAs). The optical density was measured and the ratio of the absorbance at 260 and 280 nm ranged from 1.8 to 2.0 in all samples. cDNA was generated from 1 μg of total RNA using M-MLV Reverse Transcriptase (Thermo Fisher Scientific, Waltham, MA, USA) with 1 h incubation at 37 °C. qRT-PCR was performed using the SYBR Green PCR Core Reagents Kit (Applied Biosystems, Foster City, CA, USA) as follows: one cycle at 95 °C for 10 min, 40 cycles at 95 °C for 5 s, and 60 °C for 60 s, and included no-template samples as a negative control. Real-time detection of SYBR Green fluorescence was conducted using an ABI StepOnePlus Real-Time PCR System (Applied Biosystems). Glyceraldehyde-3-phosphate dehydrogenase (*GAPDH*) mRNA (TaqMan, *GAPDH* control reagents, Applied Biosystems) was quantified as an endogenous control in each sample for normalization [16]. The qRT-PCR reactions in each sample were performed in triplicate. The relative copy number of the mRNA was calculated in reference to standard curves (cloned 10^1^ ~ 10^7^ amplicons) established by our laboratory. The expression level of each sample is presented as the value of the *GPR155* amplicon divided by that of *GAPDH* (Additional file 1: Table S1) [17].

### Bisulfite sequence analysis

We conducted methylation analysis assuming the existence of DNA hypermethylation because GPR155 harbors a CpG island in its promoter region. Genomic DNA of the cell lines was treated with bisulfite for bisulfite sequence analysis [18]. After PCR amplification using specific primers shown in Additional file 1: Table S1, the PCR products were purified using a MiniElute PCR Purification Kit (Qiagen, Hilden, Germany) and 5 ng of PCR products mixed with 9.6 pmol of sense primer were sent to Eurofins Genomics Co. Ltd. (Tokyo, Japan) for sequencing.

### Copy number analysis

Using purified genomic DNA obtained from HCC cell lines, DNA copy numbers were determined by the TaqMan Copy Number Assays (Applied Biosystems) to explore regulatory mechanisms of *GPR155* expression other than DNA methylation. A total of 20 ng of genomic DNA was amplified with specific primer pairs according to the manufacturer’s protocol using an ABI StepOnePlus Real-Time PCR System (Applied Biosystems). Three assays were employed: upstream (assay ID: Hs01092594_cn, location: Chromosome 2, 175,351,658 in the exon 1 of GPR155 gene), midstream (assay ID: Hs01971174_cn, location: Chromosome 2, 175,335,170 in the exon 6 of GPR155 gene), and downstream (assay ID: Mn00059996_cn, location: Chromosome 2, 73,351,855 at overlaps intron 14 and exon 14 of GPR155 gene). Data were analyzed using CopyCallerTM Software (Life Technologies, Carlsbad, CA, USA) [19].

### Immunohistochemical staining of GPR155 protein

Immunohistochemical staining was performed to determine the difference in GPR155 protein expression between HCC tissue and non-cancerous tissues in 60 specimens. Sections were incubated for 16 h at 4 °C with a rabbit polyclonal antibody raised against GPR155 (sc-137,511, Santa Cruz Biotechnology Inc., Dallas, TX, USA) diluted 1:200 in Antibody Diluent (Dako, Carpinteria, CA, USA). Sections were washed with phosphate buffered saline, followed by a 10-min incubation with biotinylated secondary antibody (SignalStain® Boost IHC Detection Reagent labelled by HRP, Cell Signaling Technology, Beverly, MA, USA). Antigen-antibody complexes were visualized by exposure of liquid 3, 3′-diaminobenzidine (Nichirei, Tokyo, Japan) for five minutes. Two independent observers evaluated the specimens in a blinded manner as follows: HCC > non-cancerous component, equivalent, or HCC < non-cancerous component [20].

### Statistical analysis

Differences between data of two groups were evaluated using the Mann–Whitney test. The χ2 test was used to analyze the significance of the association between the expression levels of *GPR155* mRNA and patients’ clinicopathologic parameters. Survival rates were calculated using the Kaplan–Meier method and differences in survival curves were evaluated using the log-rank test. All statistical analyses were performed using JMP 10 software (SAS Institute Inc., Cary, NC). A *p* value <0.05 was considered statistically significant.

## Results

### Expression, methylation, and copy number alteration of *GPR155* in cell lines

**Fig. 1:**
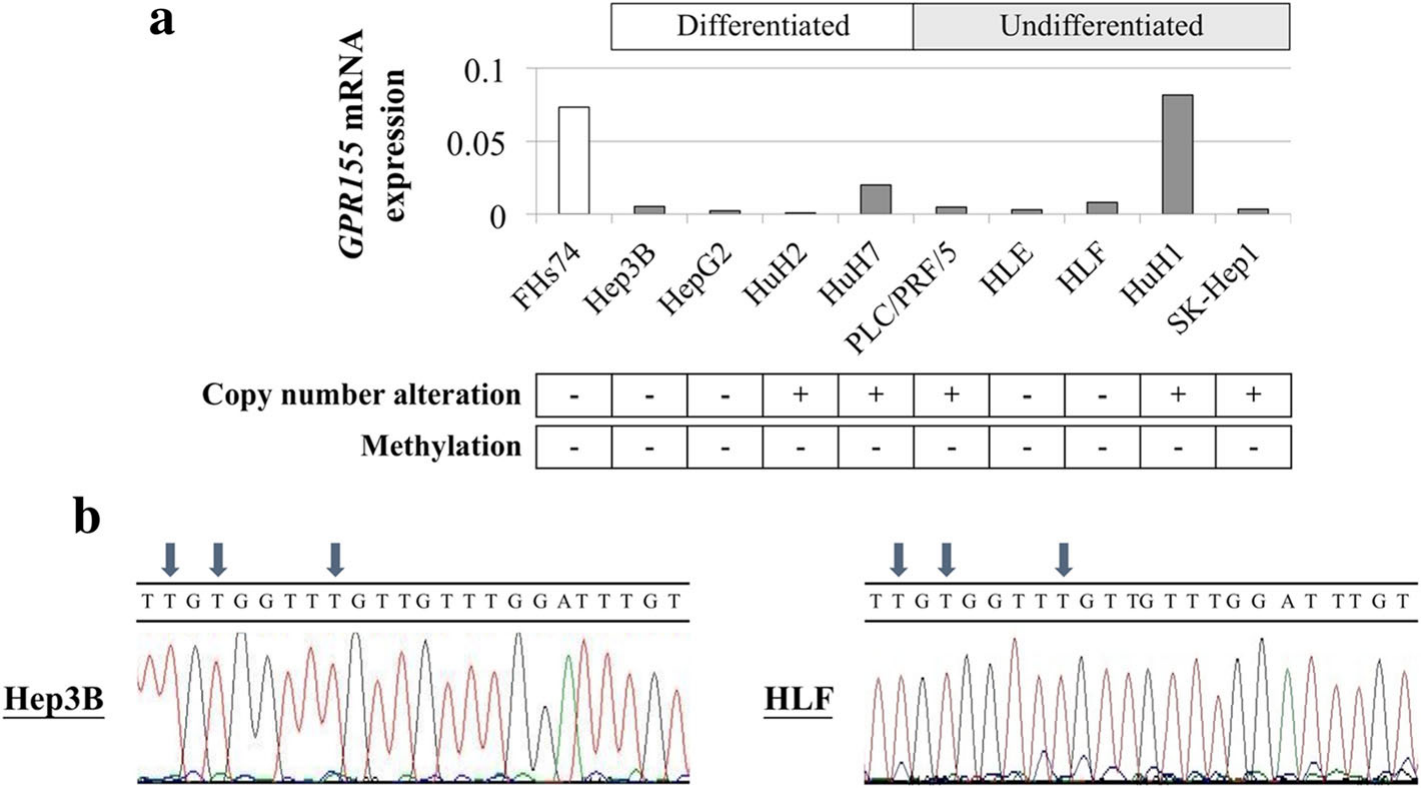
Analysis of expression, methylation, and copy number of *GPR155* in cell lines. **a** *GPR155* mRNA expression levels in HCC cell lines. Copy number alterations and methylation status of the *GPR155* promoter are summarized in lower boxes. **b** Representative results of bisulfite sequence analysis. All CpG sites were converted to TG

*GPR155* showed differential mRNA expression with decreased levels of expression in all HCC cell lines except for HuH1 compared with the control non-tumorigenic cell line FHs74 (Fig. 1a). No significant difference in expression levels of *GPR155* mRNA was observed between differentiated and undifferentiated types. Bisulfite sequence analysis revealed no DNA methylation at the region amplified by our primers within the promoter of GPR155 gene (Fig. 1b). However, copy number alterations were detected in HuH2, HuH7, PLC/PRF/5, HuH1, and SK-Hep1 cells (Fig. 1a).

### Patient characteristics

The age of the 144 patients ranged from 34 to 84 years (median 65.5 years) and the male:female ratio was 121:23. Thirty-seven patients were infected with hepatitis B virus (HBV) and 80 patients with hepatitis C virus (HCV). The number of patients with normal liver, chronic hepatitis, and cirrhosis was 10, 82, and 52, respectively. Ninety, 37, and 17 patients were in stage I, II, or III, respectively, according to the Union for International Cancer Control (UICC) classification.

### Analysis of *GPR155* mRNA and protein expression in HCC tissues

**Fig. 2:**
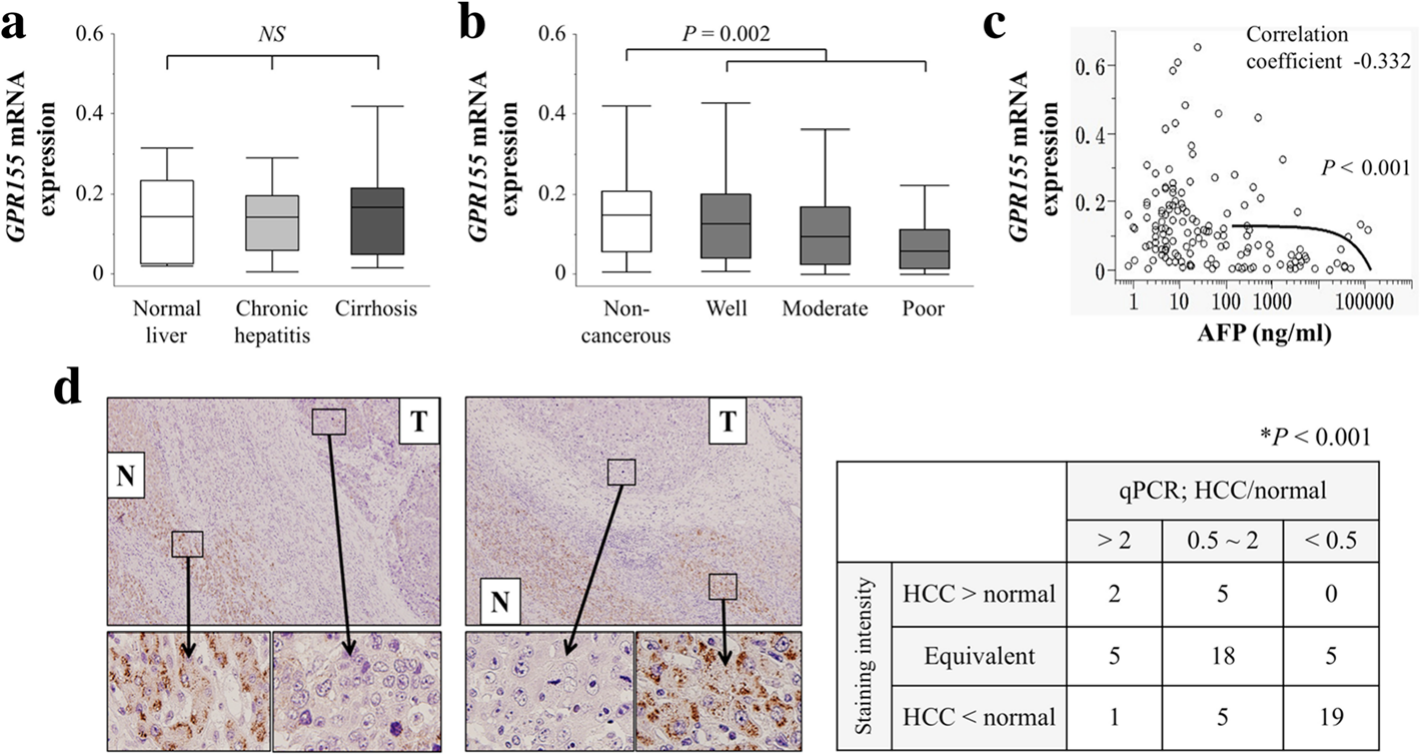
Analysis of *GPR155* expression in clinical specimens. **a** There were no significant differences in *GPR155* mRNA levels among non-cancerous tissues categorized by background uninvolved liver status. **b** *GPR155* mRNA was expressed at lower levels in HCC tissues compared with corresponding non-cancerous tissues. **c** Correlation of *GPR155* mRNA expression levels in HCC tissues with preoperative serum α-fetoprotein levels. **d** Detection of GPR155 protein in two representative patients. In both cases, cancerous tissues exhibited reduced expression compared with adjacent non-cancerous tissues (100× and 400× magnification). N, non-cancerous component; T, tumor component. A significant correlation between staining intensity and transcription patterns of *GPR155* was observed

*GPR155* mRNA expression levels in non-cancerous tissues were comparable among patients with normal liver, chronic hepatitis, and cirrhosis (Fig. 2a). HCC tissues had significantly lower expression levels of *GPR155* mRNA than the corresponding normal liver tissues (Fig. 2b). An inverse correlation between *GPR155* expression levels in HCC tissues and preoperative serum α-fetoprotein was observed (Fig. 2c). The expression patterns of *GPR155* protein were evaluated using immunohistochemical staining and two representative patients with reduced expression of GPR155 protein in the cytoplasm of cancer cells compared with non-cancerous cells are shown in Fig. 2d. The pattern of staining intensity of GPR155 protein between HCC and normal components was significantly associated with the qRT-PCR data (*p* < 0.001, Fig. 2d).

### Clinical implications of *GPR155* expression levels

**Fig. 3:**
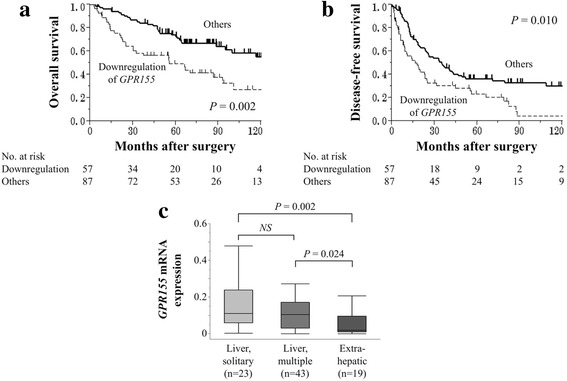
**a** Correlation between *GPR155* expression and overall survival of patients with HCC. Overall survival of patients with downregulation of *GPR155* was significantly shorter than that of patients without downregulation. **b** Correlation between *GPR155* expression and recurrence-free survival of patients with HCC. **c** *GPR155* mRNA levels in HCC tissues categorized by the initial recurrence pattern

Patients were categorized into two groups according to *GPR155* expression level. Downregulation of *GPR155* was defined as *GPR155* expression level in HCC tissue ≤50% of that in the corresponding non-cancerous tissue. Downregulation of *GPR155* was significantly associated with female sex, Pugh-Child’s classification B, α-fetoprotein >20 ng/mL, protein induced by vitamin K antagonists II >40 mAU/mL, poor differentiation, serosal infiltration, formation of capsule, infiltration to capsule, septum formation, vascular invasion, and advanced UICC stage (Table 1). The overall survival of patients with downregulation of *GPR155* was significantly shorter than that of patients without downregulation of *GPR155* (5-year survival rates 52% versus 72%, respectively, Fig. 3a). Disease-free survival was also shorter in patients with downregulation of *GPR155* than in those without (2-year disease-free survival rates 41% versus 59%, respectively, Fig. 3b). Multivariable analyses were performed for both overall and disease-free survival and downregulation of *GPR155* was not identified as an independent prognostic factor (Additional file 2: Table S2 and Additional file 3: Table S3).

**Table 1:** Association between expression level of *GPR155* mRNA and clinicopathological parameters in 144 patients with hepatocellular carcinoma

Clinicopathological parameters	Downregulation of *GPR155* (*n* = 57)	Others (*n* = 87)	*p* value
Age			0.349
< 65 year	23	42	
≥ 65 year	34	45	
Gender			0.013*
Female	4	19	
Male	53	68	
Background liver			0.188
Normal liver	2	8	
Chronic hepatitis	37	45	
Cirrhosis	18	34	
Pugh-Child’s classification			0.044*
A	50	84	
B	7	3	
Hepatitis virus			0.757
Absent	9	18	
HBV	15	22	
HCV	33	47	
AFP (ng/ml)			0.002*
≤ 20	22	56	
> 20	35	31	
PIVKA II (mAU/ml)			0.002*
≤ 40	14	44	
> 40	43	43	
Tumor multiplicity			0.078
Solitary	40	72	
Multiple	17	15	
Tumor size			0.120
< 3.0 cm	14	32	
≥ 3.0 cm	43	55	
Differentiation			0.009*
Well	7	28	
Moderate	43	55	
Poor	7	4	
Growth type			0.495
Expansive growth	46	74	
Invasive growth	11	13	
Serosal infiltration			0.031*
Absent	49	60	
Present	23	12	
Formation of capsule			<0.001*
Absent	33	76	
Present	24	11	
Infiltration to capsule			0.035*
Absent	20	46	
Present	37	41	
Septum formation			0.036*
Absent	14	36	
Present	43	51	
Vascular invasion			<0.001*
Absent	34	74	
Present	23	13	
UICC pathological stage			<0.001*
I	25	65	
II	22	15	
III	10	7	

*Abbreviations: HBV* hepatitis B virus, *HCV* hepatitis C virus, *AFP* α-fetoprotein, *PIVKA* protein induced by vitamin K antagonists, *UICC* Union for International Cancer Control. *Statistically significant difference (*p* < 0.05)

**Fig. 4:**
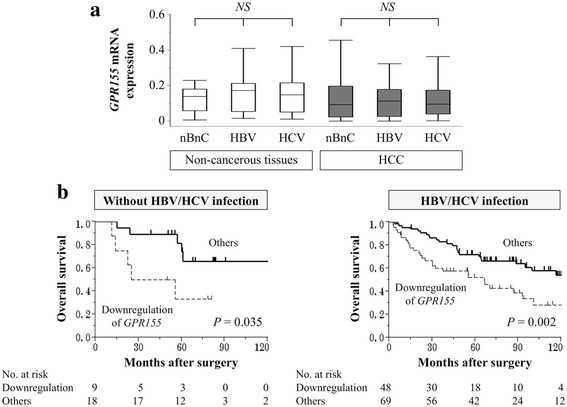
**a** Analysis of *GPR155* mRNA expression levels according to hepatitis virus infection. **b** Patients with downregulation of *GPR155* had significantly shorter overall survival in both the nonBnonC and HBV/HCV groups

We next evaluated correlations between *GPR155* expression and site of the initial recurrence. The mean *GPR155* expression level was significantly lower in patients who experienced extrahepatic (distant) recurrences compared with those with intrahepatic (liver confined) recurrences (Fig. 3c). Similar expression levels of *GPR155* mRNA were observed in both HCC and corresponding non-cancerous tissues according to the infectious status of hepatitis viruses (Fig. 4a). Patients with downregulation of *GPR155* were more likely to have a shorter overall survival than those without in patient subsets with and without HBV/HCV infection (Fig. 4b).

## Discussion

In the present study we evaluated the expression of *GPR155* and its predictive value in HCC. The GPCR superfamily of membranous receptors, of which *GPR155* is a member, has a variety of roles in intracellular signal transduction [21, 22]. When various ligands are recognized by GPCRs, GDP is converted to GTP and the α subunit and βγ subunit, acting as individual effector molecules, dissociate from the GPCR and are reported to be involved in multiple processes of cancer progression [11, 22, 23]. *GPR155* harbors an auxin efflux carrier domain, a pleckstrin/G protein-interacting region, and a winged helix repressor DNA-binding domain; however, the function of the receptor is poorly understood [12, 14]. There have been some reports of *GPR155* expression in mouse models, such as aberrant expression of *GPR155* in UV-induced melanoma and Huntington’s disease models [12, 24]. With respect to human neoplasms, only one microarray analysis indicated suppression of *GPR155* in thyroid tumor, and to our best knowledge this study is the first to evaluate *GPR155* expression in digestive cancers, including HCC [14].

We found that *GPR155* mRNA expression was decreased in 89% of HCC cell lines compared with the control non-tumorigenic cell line. As promoter hypermethylation is recognized as one of the prominent regulatory mechanisms of gene transcription [25], we conducted bisulfate sequence analysis to determine mechanisms of *GPR155* suppression; however, no methylation was detected at the CpG island within the promoter region of GPR155 gene in any of the cell lines tested. We then performed copy number analysis to explore an alternative mechanism of *GPR155* transcription because analysis of copy number variations on a genomic scale has been reported to be useful for assessing cancer progression and identifying congenital genetic abnormalities. Moreover, accumulating evidence indicates that loss of heterozygosity, mutations, and homozygous deletions are frequently present at human chromosome 2q31, the location of the *GPR155* gene [26, 27]. We found copy number alterations in five (56%) HCC cell lines that showed reduced expression levels of *GPR155* mRNA. These results indicated that copy number alteration might be one of the major regulatory mechanisms of *GPR155* transcription. However, some HCC cell lines with decreased *GPR155* mRNA expression did not show copy number alterations. When referring to The Cancer Genome Atlas database for HCC via the cBioPortal (http://www.cbioportal.org/), mutations and copy number alterations were found 0.8% and 2% of HCC tissues, respectively, though our data showed more frequent copy number alterations in HCC cell lines. Further investigation of other molecular modifications, such as acetylation of histone and microRNA expression, is expected to increase our understanding of *GPR155* regulation in HCC.

In clinical samples, *GPR155* mRNA levels were decreased in HCC tissues compared with the corresponding non-cancerous tissues, consistent with the results in cell lines. *GPR155* mRNA expression levels were equivalent among normal liver, hepatitis, and cirrhosis as background liver status. These findings suggested that alteration of *GPR155* expression may represent a specific event that occurs in the final stage of the initiation of HCC or during disease progression. Downregulation of *GPR155* was associated with more aggressive phenotypes of HCC, and subsequently linked to poorer postoperative survival. *GPR155* protein was successfully detected by immunohistochemical staining and we found a close correlation between *GPR155* protein and mRNA expression, which allowed us to evaluate the clinical significance of *GPR155* mRNA levels in a quantitative manner. Furthermore, this result may emphasize the clinical utility of *GPR155* because immunohistochemical staining is a convenient and popular method commonly available in most hospitals. Both liver biopsy samples and surgically-resected specimens can be applicable in this context.

HBV and HCV infection have been recognized as major causes of HCC [2, 28]. In the latest decade, the incidence of HCV-related HCC has been dramatically declining due to increased adoption of precautions and the introduction of a direct-acting anti-HCV agent [3, 29]. Accordingly, nonBnonC-HCC arising from chronic hepatic disease, including nonalcoholic steatohepatitis and nonalcoholic fatty liver disease, is becoming increasingly important in clinical practice [7, 30–32]. In this study, we conducted a subset analysis according to hepatitis virus infection. No significant differences in *GPR155* expression levels were observed among the nonBnonC, HBV, and HCV groups for both HCC and non-cancerous tissues. In previous literature it has been reported that nonBnonC-HCC is more prevalent in male patients, has relatively low transaminase levels, larger tumor size, advanced disease stage at the time of diagnosis, and a worse prognosis compared with HBV/HCV-related HCCs [7, 31, 33]. We found that the prognostic impact of *GPR155* expression was equivalent in nonBnonC and HBV/HCV-related HCCs. These findings highlight the clinical utility of *GPR155* expression as a prognosticator regardless of hepatitis virus infection.

Another notable finding of our study was that *GPR155* expression was associated not only with overall survival but also with initial recurrence patterns. The fact that downregulation of *GPR155* had a more remarkable effect on overall survival than disease-free survival motivated us to investigate the association between *GPR155* expression and initial recurrence patterns. Recurrence sites represent a serious issue in the management of HCC. In cases with liver-confined recurrences, repetition of liver resection is applicable and long-term survival can be expected [2]. In contrast, the prognosis of patients with extrahepatic recurrences is dismal due to the lack of effective systemic chemotherapy [3, 9, 34]. To date, there are no biomarkers for prediction of the recurrence patterns of HCC. Our findings indicate that physicians can make a risk stratification of distant recurrences and poor prognosis by determining the expression levels of *GPR155* using liver biopsies or surgical samples. Moreover, the expression levels of *GPR155* may serves as a biomarker to establish a criterion for determining an appropriate therapeutic strategy such as topical therapy or systemic chemotherapy. For future consideration, external validation is necessary.

This study was limited by its lack of sufficient functional analysis of *GPR155*, which tempers the conclusion that it acts as a tumor suppressor in HCC. Further studies including pathway analysis and functional analysis by forced expression experiments are expected to clarify the molecular mechanisms underlying the biological activities of *GPR155* in HCC.

## Conclusion

Taken together, our results indicate that downregulation of *GPR155* might be a prognostic factor and a predictor of initial recurrence sites, independent of hepatitis virus infection. Evaluation of *GPR155* expression might improve patient follow-up and treatment after liver resection, possibly leading to better prognosis.

## Additional files

Additional file 1: Table S1. Primers used in this study and annealing temperature (DOC 40 kb)

Additional file 2: Table S2. Prognostic factors for overall survival in 144 patients with hepatocellular carcinoma (DOC 49 kb)

Additional file 3: Table S3. Prognostic factors for disease-free survival in 144 patients with hepatocellular carcinoma (DOC 50 kb)

## Abbreviations

cDNA: complementary DNA
GPCR: G protein-coupled receptor
GPR155: G protein-coupled receptor 155
HBV: Hepatitis B virus
HCC: Hepatocellular carcinoma
HCV: hepatitis C virus
qRT-PCR: quantitative real-time reverse-transcription polymerase chain reaction
UICC: Union for International Cancer Control
