# Supplementary material for: Downregulation of GPR155 as a prognostic factor after curative resection of hepatocellular carcinoma
Source: BMC Cancer. 2017 Sep 1;17:610. doi: 10.1186/s12885-017-3629-2 (PMC5580443; doi:10.1186/s12885-017-3629-2)
Supplement: Supplementary file 1 — Primers used in this study and annealing temperature (DOC 40 kb) [file 12885_2017_3629_MOESM1_ESM.doc]

| Gene | Experiment | Type | Sequence (5´ - 3´) | Product size | Annealing temperature |
| --- | --- | --- | --- | --- | --- |
| *GPR155* | qRT-PCR | forward | AGCAAAGCTGGACTATTCCCT | 125 bp | 60 °C |
|  |  | reverse | GCCACCAAATAAATGTACTGGA |  |  |
|  | Bisulfite  sequencing 2 | forward | TTTTTGTTTTTGTTTTTTAGGTTTG | 197 bp | 60 °C |
|  |  | reverse | AACTAAAAATAACAATTCTATCTCC |  |  |
| *GAPDH* | qRT-PCR | forward | GAAGGTGAAGGTCGGAGTC | 226 bp | 60 °C |
|  |  | probe | CAAGCTTCCCGTTCTCAGCC |  |  |
|  |  | reverse | GAAGATGGTGATGGGATTTC |  |  |

**Additional file 1: Table S1.** **Primers used in this study and annealing temperature**

*Abbreviations:* *GPR155* G protein-coupled receptor 155, *GAPDH* glyceraldehyde-3-phosphate dehydrogenase, *qRT-PCR* quantitative real-time reverse-transcription polymerase chain reaction.
