# Supplementary material for: Downregulation of GPR155 as a prognostic factor after curative resection of hepatocellular carcinoma
Source: BMC Cancer. 2017 Sep 1;17:610. doi: 10.1186/s12885-017-3629-2 (PMC5580443; doi:10.1186/s12885-017-3629-2)
Supplement: Supplementary file 2 — Prognostic factors for overall survival in 144 patients with hepatocellular carcinoma (DOC 49 kb) [file 12885_2017_3629_MOESM2_ESM.doc]

**Additional file 2: Table S2. Prognostic factors for overall survival in 144 patients with hepatocellular carcinoma**

| **Variable** | **n** | **Univariate** | | | **Multivariate** | | |
| --- | --- | --- | --- | --- | --- | --- | --- |
|  |  | **Hazard ratio** | **95% CI** | ***P* value** | **Hazard ratio** | **95% CI** | ***P* value** |
| Age (≥ 65) | 79 | 1.75 | 0.96 – 3.30 | 0.068 |  |  |  |
| Gender (male) | 121 | 1.82 | 0.78 – 5.29 | 0.178 |  |  |  |
| Background liver (cirrhosis) | 52 | 1.53 | 0.84 – 2.75 | 0.161 |  |  |  |
| Pugh-Child’s classification (B) | 10 | 1.68 | 0.50 – 4.19 | 0.360 |  |  |  |
| AFP (> 20 ng/ml) | 66 | 1.96 | 1.09 – 3.58 | 0.024 | 1.19 | 0.70 – 2.03 | 0.517 |
| PIVKA II (> 40 mAU/ml) | 86 | 1.90 | 1.03 – 3.71 | 0.041 | 1.64 | 0.87 – 3.20 | 0.128 |
| Tumor multiplicity (multiple) | 32 | 1.83 | 0.94 – 3.38 | 0.073 |  |  |  |
| Tumor size (≥ 3.0 cm) | 98 | 2.84 | 1.38 – 6.64 | 0.004 | 1.01 | 0.55 – 1.93 | 0.972 |
| Tumor differentiation (well) | 35 | 0.72 | 0.34 – 1.41 | 0.349 |  |  |  |
| Growth type (invasive growth) | 24 | 1.71 | 0.84 – 3.26 | 0.136 |  |  |  |
| Serosal infiltration | 35 | 2.23 | 1.16 – 4.11 | 0.017 | 2.17 | 1.22 – 3.79 | 0.009 |
| Formation of capsule | 97 | 0.95 | 0.52 – 1.81 | 0.861 |  |  |  |
| Infiltration to capsule | 78 | 1.24 | 0.69 – 2.29 | 0.478 |  |  |  |
| Septum formation | 94 | 0.77 | 0.43 – 1.43 | 0.402 |  |  |  |
| Vascular invasion | 36 | 3.75 | 2.05 – 6.78 | < 0.001 | 1.52 | 0.83 – 2.74 | 0.173 |
| Downregulation of *GPR155* | 57 | 2.46 | 1.49 – 4.06 | <0.001 | 1.43 | 0.79 – 2.56 | 0.232 |

*Abbreviations:* *CI* confidence interval, *AFP* α-fetoprotein, *PIVKA* protein induced by vitamin K antagonists.
