# Supplementary material for: Downregulation of GPR155 as a prognostic factor after curative resection of hepatocellular carcinoma
Source: BMC Cancer. 2017 Sep 1;17:610. doi: 10.1186/s12885-017-3629-2 (PMC5580443; doi:10.1186/s12885-017-3629-2)
Supplement: Supplementary file 3 — Prognostic factors for disease-free survival in 144 patients with hepatocellular carcinoma (DOC 50 kb) [file 12885_2017_3629_MOESM3_ESM.doc]

**Additional file 3: Table S3. Prognostic factors for disease-free survival in 144 patients with hepatocellular carcinoma**

| **Variable** | **n** | **Univariate** | | | **Multivariate** | | |
| --- | --- | --- | --- | --- | --- | --- | --- |
|  |  | **Hazard ratio** | **95% CI** | ***P* value** | **Hazard ratio** | **95% CI** | ***P* value** |
| Age (≥ 65) | 79 | 1.20 | 0.81 – 1.78 | 0.367 |  |  |  |
| Gender (male) | 121 | 1.92 | 1.09 – 3.70 | 0.022 | 1.38 | 0.75 – 2.73 | 0.311 |
| Background liver (cirrhosis) | 52 | 1.21 | 0.80 – 1.80 | 0.358 |  |  |  |
| Pugh-Child’s classification (B) | 10 | 1.56 | 0.70 – 3.03 | 0.255 |  |  |  |
| AFP (> 20 ng/ml) | 66 | 1.35 | 0.91 – 2.00 | 0.139 |  |  |  |
| PIVKA II (> 40 mAU/ml) | 86 | 1.58 | 1.05 – 2.39 | 0.026 | 1.21 | 0.78 – 1.89 | 0.388 |
| Tumor multiplicity (multiple) | 32 | 1.50 | 0.94 – 2.33 | 0.089 |  |  |  |
| Tumor size (≥ 3.0 cm) | 98 | 1.47 | 0.96 – 2.29 | 0.075 |  |  |  |
| Tumor differentiation (well) | 35 | 0.85 | 0.53 – 1.33 | 0.486 |  |  |  |
| Growth type (invasive growth) | 24 | 1.14 | 0.66 – 1.89 | 0.614 |  |  |  |
| Serosal infiltration | 35 | 2.38 | 1.52 – 3.63 | <0.001 | 1.94 | 1.21 – 3.03 | 0.006 |
| Formation of capsule | 97 | 1.36 | 0.89 – 2.12 | 0.160 |  |  |  |
| Infiltration to capsule | 78 | 1.24 | 0.84 – 1.84 | 0.288 |  |  |  |
| Septum formation | 94 | 0.87 | 0.58 – 1.31 | 0.498 |  |  |  |
| Vascular invasion | 36 | 2.40 | 1.52 – 3.69 | <0.001 | 1.84 | 1.14 – 2.89 | 0.014 |
| Downregulation of *GPR155* | 57 | 1.87 | 1.25 – 2.77 | 0.002 | 1.29 | 0.83 – 2.00 | 0.248 |

*Abbreviations:* *CI* confidence interval, *AFP* α-fetoprotein, *PIVKA* protein induced by vitamin K antagonists.
